# The Book World of Medicine and Science

**Published:** 1896-04-25

**Authors:** 


### The Book World of Medicine and Science.

The Schott Methods of Treatment of Chronic Diseases of the Heart. By W. Bezly Thorne, M.D. (London: J. and A. Churchill. 1896. 8vo., 5s.)

The Schott treatment lay quite dormant in England, or rather it was not heard of until Dr. Bezly Thorne brought it under notice, with the happy result that a real interest has been awakened, and the book named at the head of this column is already in a second edition, although the first was only published in 1895. Dr. Bezly Thorne gives us in small compass, and in a most readable form, a complete description of the Schott methods; the composition of the waters; the various modes of administering the baths; and the gymnastic exercises which are always used concomitantly with the baths. Dr. Thorne further shows—and we are glad to have his testimony on the point—that precisely similar effects may be looked for in England from baths artificially prepared as are obtained in Nauheim from the natural waters. This fact is a most important one, and it greatly extends the usefulness of the treatment, as it places it within reach of everyone. Indeed, some of Dr. Thorne's own observations were made at Llangammarch Wells. Dr. Thorne by no means neglects methods other than the baths and gymnastics, for we find him expressly pointing out the value of a careful dietary as an adjuvant, and, above all, the importance of water, which "of all internal remedies for cardiac affections generally, aneurism not excepted, is perhaps the most powerful." At the end of the book details of several cases are given, and these must seem very wonderful to practitioners who have never seen the Schott treatment, but many parallel cases could be adduced by those who have adopted it in England or studied it at Nauheim. The methods are surely taking an important place in our mode of treating heart diseases, and the practitioner will find this volume a safe guide.

Comparative Embryology: Text-book of the Embryology of Invertebrates. By Korschelt and Heider: Translated from the German by Professor E. L. Mark and Mr. W. McM. Woodworth, with additions by the Authors and Translators. Part I. Porifera, Cnidaria, Ctenophora, Vermes Enteropneust Echinodermata. (London: Swan Sonnenschein and Co., 1895.)

The specialisation of science, inevitable as it was, brought with it one disadvantage for the student of medicine. In the old days, investigation of the zoological sciences was almost entirely in the hands of doctors, and the medical student, along with his training in his craft, was led to philosophical views of the whole living world. The modern student is taught chiefly by professional experts who have had to abandon pure science, and by scientific specialists who are ignorant of the professional needs of medical students. We fear that this excellent embryology, as it deals chiefly with animals that have no place in the narrow limits of the curricula devised for medical students, will come the way of few of them. It is the more to be regretted, as the advances in embryology have shown clearly that the developmental history of so specialised a type as man can be explained and understood only by the light of comparative embryology in its widest sense. Take, for instance, the early stages in the development of the human ovum. By themselves they are inexplicable. But when it is seen that the changes are partly memories of ancestral modes of development, and partly adaptations to special conditions, the confused facts arrange themselves in an orderly plan. At one time, the eggs of mammalia possessed an abundant supply of food-yolk to nourish the embryo, which developed outside the body of the mother. When the eggs came to be retained within the body of the mother and nourished directly from her blood, the supply of yolk no longer was provided. But legacies from the old condition were retained in the formation of the early stages of the embryo, and these again were modified to serve for attachment to the wall of the uterus. In many different groups of the animal kingdom the same series of changes are to be seen. This case is only one of many illustrations that might be chosen of the fashion in which comparative embryology illuminates the obscure problems of human embryology. Drs. Korschelt and Heider are both well-known authorities, and their volume shows a wide knowledge and considerable care in the selection and criticism of a wide literature. Since Balfour wrote his classical treatise an enormous quantity of additional information has been gained, and the best of this is incorporated in the present volume. We miss, however, the wonderful breadth of view and masterly power of generalisation that will preserve Balfour's book long after the facts in it are much more out of date than is yet the case. The additions to embryology since his time have been chiefly minor alterations and additions to the great edifice his genius constructed, not reconstructions from new designs. As the authors and translators profess to have made additions and corrections since the original German edition appeared, we think it is to be regretted that they have not modified their somewhat cavalier treatment of the systems of cavities found in the cœlomate animals. It is one of the most interesting points of recent embryological morphology, and they have been content with a short account of views that have been long since abandoned by most zoologists. The actual translation is well done, although we think that the translators might have found a better word for the German "anlage" than the humorous English equivalent they employ. The printing and the process-block illustrations are very clear and good.

The Medical Register and the Dentists' Register for 1896. (Printed and published under the direction of the General Council of Medical Education and Registration of the United Kingdom. London: Spottiswoode and Co., 54, Gracechurch-street, E.C.)

Mr. Miller, the registrar, is to be congratulated upon the prompt publication of these registers, which are now more complete than ever before. It is interesting to note that, whereas in 1876 the number of new names added by registration to the Medical Register was 1,009, in 1896 the number had increased to 1,446. The average addition by registration for the last 26 years has been 1,268, and during the last five years 1,462 names. Five hundred and thirty-eight names have been removed owing to death, which is a number slightly above the average of the last five years, *i.e.*, 536. In addition to the mere names, the register now contains summaries of the principal medical and other Acts, and various tables and items of information which cannot fail to be of importance and interesting to medical practitioners generally.

